# Hospitalisation among immigrants in Italy

**DOI:** 10.1186/1742-7622-3-4

**Published:** 2006-05-11

**Authors:** Laura Cacciani, Giovanni Baglio, Lorenza Rossi, Enrico Materia, Maurizio Marceca, Salvatore Geraci, Angela Spinelli, John Osborn, Gabriella Guasticchi

**Affiliations:** 1 Agency for Public Health of Lazio Region, Rome, Italy; 2 Caritas Diocesana of Rome, Rome, Italy; 3 Department of Public Health Sciences, University of Rome "La Sapienza", Rome, Italy

## Abstract

**Background:**

Immigration is increasing in Italy. In 2003, 2.6 million foreign citizens lived in the country; 52% were men and the majority were young adults who migrated for work. The purpose of this study was to investigate differences in hospitalisation between immigrants and the resident population during the year 2000 in the Lazio region.

**Methods:**

Hospital admissions of immigrants from Less Developed Countries were compared to those of residents. We measured differences in hospitalisation rates and proportions admitted.

**Results:**

Adult immigrants have lower hospitalisation rates than residents (134.6 vs. 160.5 per thousand population for acute care; 26.4 vs. 38.3 for day care). However, hospitalisation rates for some specific causes (injuries, particularly for men, infectious diseases, deliveries and induced abortions, ill-defined conditions)  were higher for immigrants than for residents. Immigrants under 18 years seem to be generally healthy; causes of admission in this group are similar to those of residents of the same age (respiratory diseases, injuries and poisoning). The only important differences are for infectious and parasitic diseases, with a higher proportion among immigrant youths.

**Conclusion:**

The low hospitalisation rates for foreigners may suggest that they are a population with good health status. However, critical areas, related to poor living and working conditions and to social vulnerability, have been identified. Under-utilisation of services and low day care rates may be partially due to administrative, linguistic, and cultural barriers. As the presence of foreigners becomes an established phenomenon, it is important to evaluate their epidemiological profile, develop instruments to monitor and fulfil their specific health needs and plan health services for a multi-ethnic population.

## Introduction

Immigration in Italy is relatively recent, constantly increasing, and nowadays accepted as an intrinsic and widespread phenomenon of the demographic and social dynamics of the nation. A positive migratory balance was observed in 1981, and by 2000 there were about 1.35 million adult immigrants legally residing in the country [1]. Following the latest legislation on immigration (law n.189/2002), this number reached 2.2 million in 2003, together with an additional 400,000 individuals aged under 18 years who do not have their own residence permits. About 52% are men and the majority of immigrants are young adults.

Immigrants in Italy come from many different areas, but mainly from Less Developed Countries (LDCs, see endnote 1). In recent years, there has been a more rapid increase in immigration from Central-Eastern Europe, which now accounts for 60% of the formal requests for permission to stay, and is the main area of origin of foreigners living in Italy, followed by North Africa [2].

As the number of immigrants continues to increase, it becomes even more important to evaluate their impact on the socio-cultural, economic and health fabric of the country, and to promote adequate programmes and policies. With regard to health, it is important to discover their epidemiological profile and to investigate their access to health services (see endnote 2), in order to identify and monitor their health needs, and to remove barriers to health care. Migrants may constitute a risk group and should have specific targets for health policy.

Some studies have suggested that social and economic inequalities are fundamental causes of ethnic health inequalities [3,4]. Conversely, the impact of socio-economic status on health could differ between ethnic groups [5]. Being an immigrant may have an influence on health through complex mechanisms, encompassing genetic, social, economic and cultural elements [6], and requiring specific investigation.

The epidemiological profile of a foreign population could be investigated by comparing their utilisation of health care services with that of the indigenous population. Different patterns of utilisation have been identified in several studies. A Danish study [7] showed that duration of hospital stay is longer for foreigners than for residents for some diagnoses but shorter for others, although no overall effect was found. In The Netherlands [8], it has been suggested that immigrants have an epidemiological profile similar to disadvantaged Dutch, although the prevalence of some infectious diseases and child mortality rates are higher among Turkish and Moroccan immigrants. Another study from The Netherlands [9] reported a lower use of specialised health care among immigrants, possibly due to difficulties of access. However, a study on health status and hospital utilisation by recent immigrants to New York City [10] concluded that foreign-born people living there appeared to be healthier and consumed fewer hospital resources than U.S.-born populations.

In the Italian context, three major aspects are worth mentioning as potential obstacles to meeting the needs of the foreign population. Firstly, the migrant population in Italy is characterised by rapid transformation; its health needs are mutable and difficult to identify. Usually, migrants have come to Italy for work or to be reunited with their families, and, more recently, to seek asylum. Secondly, immigrants in Italy show high levels of continued mobility through both internal and external migration, which hinders the possibility of following their health status over time and distinguishing between acquired and imported causes of illness. Finally, a substantial number of immigrants do not hold a legal residence permit; this group probably comprises individuals with different health needs [11].

At the national level, studies have been conducted on migrants' health [12-18], some using administrative data, others through specific surveys. Nevertheless, additional evidence on the epidemiological profile of the immigrant population residing in our country is necessary due to its continuous increase and evolution. The aim of this study was to identify the pattern of hospital use among immigrants living in Lazio, Italy. The analysis of hospital discharge data has great potential to identify health needs and the special problems faced by immigrants. We have analysed data from Lazio, which is the region with the second largest number of immigrants: in 2003, 330,695 foreign citizens were living in the region, representing about 6% of the resident population, and 15% of the total foreign population living in Italy [2]. The hospitalisation pattern among foreigners was compared with that of the total regional population.

## Subjects and methods

The study is based on discharges from hospitals collected in Italy by the Italian Hospital Information System for the year 2000. In particular, in Lazio, this system has reached a high level of validity and completeness, and there is no evidence to suggest that the quality of these data differs between foreigners and the general population. We defined immigrants, foreign citizens or foreigners as people without Italian citizenship. Immigrants can be granted Italian citizenship only in very restricted circumstances – for example, having an Italian parent, marriage to an Italian or after at least ten years' legal residence.

We analysed all acute and day care discharges from all hospitals in Lazio. We identified the immigrants admitted and the immigrant population from their stated citizenship. Data on patients' migration across regional boundaries among immigrants have not been included. However, within relatively short periods of time they probably represent a small percentage (estimated at less than 3%) of total discharges of immigrants in 2000.

Two analyses were performed. The first included only the adult population (aged 18 years or above) and, for the immigrants, only those coming from LDCs. Crude, age-specific, and age- and gender-standardised hospitalisation rates, as well as proportions of admissions separately by gender, setting of care (acute or day care), and International Classification of Diseases Ninth Revision, Clinical Modification (ICD-9-CM) main groups of diagnoses [19], were calculated in the immigrant population. The denominators of the rates for immigrants were based on data issued by the Ministry of the Interior as of 1st January 2000. Such figures rely on the so-called "stay permit", granted to immigrants to enable them to live legally in the country. They include information on age, gender and country of origin, which allowed us to calculate rates for immigrants. Unfortunately, since immigrants who do not hold a stay permit (irregular population) are not included in this denominator, and it is very difficult to estimate this number, we have necessarily had to use an under-estimate of the population to calculate both specific and standardised rates. We attempted to verify the validity of standardised rates by a method of standardisation that we call "in direct-in verse standardisation" (see endnote 3).

During the year 2000, about 242,000 immigrants were legally living in Lazio, representing less than 5% of the resident population in the region, and 18% of the immigrant population living in Italy. Of these, 189,905 were adults from LDCs. Hospitalisation rates for immigrants were compared with those for the region as a whole. The denominator of the rates for the total region was the resident population as of 1st of January 2000, published by the National Institute of Statistics (see endnote 4). These data were also used as the reference population to standardise rates. Rate ratios (RR, immigrants versus total regional population) for selected groups of diagnoses were also calculated together with 95% confidence intervals (CIs), using the log-transform at ion of the rate [20].

The second analysis was conducted for the population under 18; we did not calculate rates because no reliable data of the immigrant population by age are available. In fact, some of these minors are included in the stay permits of their parents. We compared the percentage distribution of discharges by ICD-9-CM main group of diagnoses between the foreign citizens born abroad, foreign citizens born in Italy and Italian citizens.

SAS System for Windows release 8.02 was used for the statistical analysis.

## Results

During the year 2000 there were 283,985 immigrants discharged from hospitals in Italy (2.2% of the total). Table 1 shows the regional distribution of hospital discharges of immigrants in Italy. There is great variability between regions (range 0.4% to 4.6%), with higher values in central-northern regions. In Lazio 36,135 hospital discharges were of immigrants (3.1% of all hospital discharges). This figure is 12.7% of all hospital discharges of immigrants in Italy.

**Table 1:** Hospital discharges among foreigners in Italy, 2000

Regions	Discharges of foreigners		Percentage of total hospitalisation
		
	N	%	
North	178119	62.7	3.1
Piemonte	24978	8.8	3.0
Valle d'Aosta	640	0.2	2.8
Lombardia	70049	24.7	3.3
Bolzano	4842	1.7	4.6
Trento	3386	1.2	3.2
Veneto	30048	10.6	3.1
Friuli Venezia Giulia	5780	2.0	2.5
Liguria	11486	4.0	2.8
Emilia Romagna	26910	9.5	2.8
			
Center	72837	25.6	3.0
Toscana	24866	8.8	3.4
Umbria	6151	2.2	3.0
Marche	5685	2.0	1.8
Lazio	36135	12.7	3.1
			
South and Islands	33029	11.6	0.7
Abruzzo	3067	1.1	0.9
Molise	264	0.1	0.4
Campania	11093	3.9	0.9
Puglia	5349	1.9	0.5
Basilicata^a^			
Calabria	2534	0.9	0.6
Sicilia	7586	2.7	0.7
Sardegna	3136	1.1	0.9
			
Total	283985	100.0	2.2

a: Not available

Sources: Italian Ministry of Health

### Adult population (18+ years old)

We analysed 25,451 acute care hospitalisations (20,419 acute and 5032 day care admissions) of immigrants from LDCs aged 18 years or more, representing approximately 3% of all discharges in the region. Overall, immigrants have lower age- and gender-standardised hospitalisation rates compared with the total resident population, both for acute (134.6, (95% Cl: 133.0 – 136.0) vs 160.5 (95% Cl: 160.1 – 160.9) per 1000 population) and day care admissions (26.4 (95% Cl: 25.6 – 27.1) vs. 38.3 (95% Cl: 38.1 – 38.5) per 1000).

When we re-calculated age- and gender-standardised hospitali sat ion rates using the method of indirect-inverse standardisation (see endnote 3), we observed that rate ratios changed from 0.84 to 0.95 for acute care and from 0.70 to 0.81 for day care, under the assumption of no irregular immigration; and from 0.84 to 0.79 and from 0.70 to 0.68 assuming 20% irregular immigration out of total observed immigrant population (see endnote 5).

The age and gender distribution of the hospitalised foreign population reflects the demographic profile of the migrant population in the region, which shows larger proportions in younger groups (Table 2). The highest percentages of acute episodes of care relate to the 18 to 34 year age group: 61.5% for women and 45.2% for men. Day care in this group is even higher for females (68.9%). The overall percentage of hospitalisation in the group aged over 65 years is negligible.

**Table 2:** Number and percentage of hospital discharges by age, setting of care, and gender. Foreign citizens from Less Developed Countries and residents, Lazio, 2000

Age groups (years)	Acute care				Day care				Foreign population	
			
	Foreigners		Residents		Foreigners		Residents			
					
	N^a^	%^b^	N^a^	%^b^	N^a^	%^b^	N^a^	%^b^	N^a^	%^b^
	Males									
18–34	3428	45.2	46151	14.9	395	41.6	11494	15.8	53023	53.9
35–49	2677	35.3	48399	15.6	337	35.5	14051	19.3	35613	36.2
50–64	864	11.4	76983	24.8	131	13.8	20281	27.9	7122	7.2
65+	611	8.1	139238	44.8	87	9.2	26931	37.0	2700	2.7
										
Total	7580	100.0	310771	100.0	950	100.0	72757	100.0	98458	100.0
	Females									
18–34	7893	61.5	96888	24.9	2812	68.9	22126	23.5	47239	51.7
35–49	3249	25.3	69353	17.9	996	24.4	21811	23.2	31936	34.9
50–64	983	7.7	70787	18.2	171	4.2	23079	24.6	9329	10.2
65+	714	5.6	151421	39.0	103	2.5	26955	28.7	2943	3.2
										
Total	12839	100.0	388449	100.0	4082	100.0	93971	100.0	91447	100.0

a: Frequency

b: Column percentage

**Figure 1:**
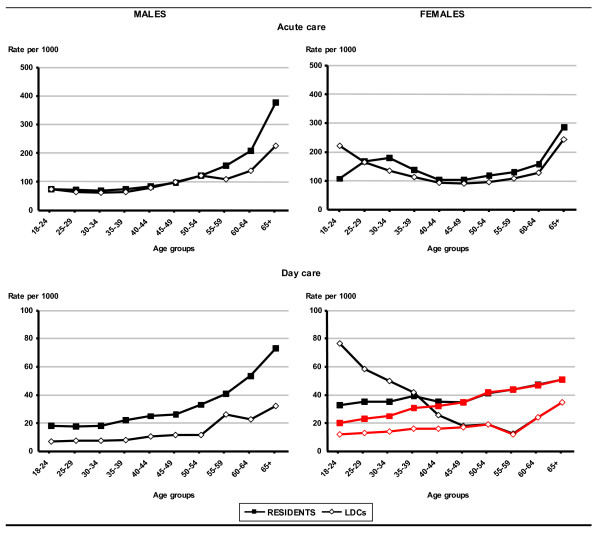
Age-specific hospitalisation rates by gender and setting of care. Foreign citizens from Less Developed Countries (LDCs) and residents, age 18+, Lazio, 2000. (Red lines indicate trends excluding induced abortions).

The age-specific hospitalisation rates by gender and setting of care (Figure 1) show a very similar pattern for foreigners and the comparison group, around 100 per 1000 population for acute admissions of males up to the age group 50–54. Starting from this age, rates for residents show a remarkable increase, whereas rates for immigrants increase modestly. Women show a very different pattern at reproductive ages. The peak in admissions is delayed for resident women, probably because the majority of births in this group occur at older ages compared with women coming from LDCs.

Day care rates are far greater in the resident population for both men and women with the exception of women below 40 years; in this group, rates are much higher for immigrants than residents (more than double in the 18 to 24 year age group). This difference is exclusively due to induced abortions, as shown when these are excluded from the analysis (red lines in Figure 1).

Table 3 shows the absolute and relative number of acute care discharges in decreasing order of frequency, grouped by ICD-9-CM diagnosis. Around 50% of foreign male admissions are due to injuries and diseases of the digestive and circulatory systems, whereas deliveries account for 45% of all hospitalisations among foreign women.

**Table 3:** Number and percentage of hospital discharges by ICD-9-CM main group of diagnoses and gender. Acute care, foreign citizens from Less Developed Countries, age 18+, Lazio, 2000

Main group of diagnoses	ICD-9-CM	Males		Females		Total	
				
		N^a^	%^b^	N^a^	%^b^	N^a^	%^b^
Pregnancy, Childbirth, Puerperium	630–677			5775	45.0	5775	28.3
Injury and Poisoning	800–999	1796	23.7	776	6.0	2572	12.6
Digestive System	520–579	1139	15.0	877	6.8	2016	9.9
Genitourinary System	580–629	325	4.3	1051	8.2	1376	6.7
Symptoms, Signs and Ill-Defined Conditions	780–799	621	8.2	697	5.4	1318	6.5
Circulatory System	390–459	703	9.3	546	4.3	1249	6.1
Neoplasms	140–239	298	3.9	699	5.4	997	4.9
Respiratory System	460–519	583	7.7	350	2.7	933	4.6
Infectious and Parasitic Diseases	001–139	457	6.0	321	2.5	778	3.8
Mental Disorders	290–319	437	5.8	316	2.5	753	3.7
Nervous System and Sense Organ	320–389	381	5.0	344	2.7	725	3.6
Musculoskeletal System and Connective Tissue	710–739	339	4.5	298	2.3	637	3.1
Skin and Subcutaneous Tissue	680–709	188	2.5	148	1.2	336	1.6
Endocrine, Nutritional and Metabolic Diseases and Immunity Disorders	240–279	89	1.2	183	1.4	272	1.3
Congenital Anomalies and conditions in the perinatal period	740–779	39	0.5	76	0.6	115	0.6
Blood and Blood-Forming Organs	280–289	45	0.6	69	0.5	114	0.6
Other		140	1.8	313	2.4	453	2.2
							
Total		7580	100.0	12839	100.0	20419	100.0

a: Frequency

b: Column percentage

We analysed gender and age-specific hospitalisation rates (acute care only) for selected groups of diagnoses where immigrants from LDCs show greater age-specific rates of admission compared with the total resident population. Hospitalisations for injuries were higher in foreign males than resident males in all age groups (RRs 1.18 (95% Cl: 1.09 – 1.27) among 18 to 29 year olds, 1.37 (1.28 – 1.46) among 30 to 49 year olds), and 1.25 (1.01 – 1.53) among 50 to 64 year olds. Females also had a higher risk of admission for injuries in the first two age groups (RR = 1.16 and 1.20; 95% CIs: 1.02 – 1.30 and 1.07 – 1.34 respectively), and at the older ages (RR = 1.25, 95% Cl 1.22 – 1.27). About 40% of injuries were specified as fractures, for both acute and day care.

For infectious diseases, risks for young males and women were respectively 2.63 and 2.83 (95% CIs: 2.24 – 3.09 and 2.38 – 3.36) in the 18 to 29 year age group, and 1.57 and 2.25 (95% CIs 1.40 – 1.76 and 1.93 – 2.61) among those aged 30 to 49 years. About 40% of infectious diseases are HIV infections, and 20% are tuberculosis.

Young women (18–29 years) have a higher risk for causes associated with pregnancies, childbirth and puerperium, showing a RR of 1.73 (95% Cl 1.67 – 1.79). Normal deliveries account for about 30% of hospitalisations for this cause, and legally induced abortions for 37%.

Likewise, hospitalisation rates for Symptoms, Signs and Ill-Defined Conditions are higher for immigrants than residents in all age groups except females over 64, with RR of 1.34 in the 18 to 29 year age group, 1.33 among 30 to 49 year-olds, and 1.28 in those aged 50 to 64 years (95% Cl: 1.18 – 1.52, 1.19 – 1.49, 1.04 – 1.59 respectively); and higher for young males (RR = 1.24, 95% Cl: 1.06 – 1.44). Abdominal pain accounts for 24% of discharges, renal colic for 12%, and syncope and collapse for 10%.

### Youths under 18

There were 156,428 discharges of youths under 18 years of age in the Lazio region in 2002. Of these, 7325 were foreign citizens (4.7% of all discharges in this age); 4136 were born abroad and 3189 in Italy. In 2000, these numbers were 5003 (3.8%), 3069 and 1934 respectively. Almost half (44.8%) the children were citizens of Eastern European countries.

Table 4 shows, in decreasing order of frequency, the absolute and relative number of acute care discharges (grouped by ICD-9-CM main group diagnosis), by citizenship and place of birth. Diseases related to the respiratory system were the most common diagnosis among foreign citizens born abroad (14.0%), as well as among foreigners born in Italy (20.7%) and Italians (21.1%). Infectious and parasitic diseases as a cause of hospitalisation were more frequent among foreign than Italian citizens (7.0% and 9.8% vs 5.0%). The percentage of congenital anomalies was higher among the foreigners born in Italy, while the diagnoses of injury and poisoning were more frequent among Italian citizens and foreigners born abroad. However, given that the age distributions for the immigrants and Italians differ, the percentages observed for the immigrants were directly standardised using the Italian population as a standard. Generally, the result of the standardisation makes the proportions observed for the immigrants closer to those of the Italians.

**Table 4:** Number and percentage of hospital discharges by citizenship, place of birth and ICD-9-CM main group of diagnoses. Acute care, age<18 years, Lazio, 2002

Main group of diagnoses	Citizenship							
	
	Foreign						Italian	
	Born abroad			Born in Italy				
				
	N^a^	%^b^	ST%^c^	N^a^	%^b^	ST%^c^	N^a^	%^b^
Respiratory System	331	14.0	16.8	387	20.7	17.2	16847	21.1
Injury and Poisoning	311	13.2	11.7	88	4.7	5.0	9437	11.8
Symptoms, Signs and Ill-Defined Conditions	233	9.9	10.2	188	10.1	10.1	8703	10.9
Digestive System	212	9.0	9.0	153	8.2	8.6	8288	10.4
Congenital Anomalies	144	6.1	7.6	216	11.6	7.4	5556	6.9
Nervous System and Sense Organ	129	5.5	5.5	100	5.4	4.4	4647	5.8
Genitourinary System	111	4.7	4.4	103	5.5	6.7	4407	5.5
Infectious and Parasitic Diseases	165	7.0	7.6	183	9.8	8.2	4016	5.0
Other	179	7.6	6.9	109	5.8	5.5	3519	4.4
Endocrine, Nutritional and Metabolic Diseases and Immunity Disorders	100	4.2	4.3	55	2.9	3.1	2775	3.5
Musculoskeletal System and Connective Tissue	69	2.9	2.4	25	1.3	2.7	2537	3.2
Neoplasms	96	4.1	3.8	49	2.6	2.2	2330	2.9
Skin and Subcutaneous Tissue	63	2.7	2.2	39	2.1	1.5	1942	2.4
Conditions in the perinatal period	2	0.1	0.2	66	3.5	1.5	1256	1.6
Circulatory System	29	1.2	1.2	22	1.2	1.7	1225	1.5
Mental Disorders	39	1.6	1.3	25	1.3	4.7	1120	1.4
Blood and Blood-Forming Organs	53	2.2	2.1	56	3.0	3.6	1042	1.3
Pregnancy, Childbirth, Puerperium	99	4.2	2.7	4	0.2	5.8	333	0.4
								
Total	2365	100.0	100.0	1868	100.0	100.0	79980	100.0

a: Frequency

b: Column percentage

c: Age-standardised proportion (directly standardised using the Italians as a standard)

## Discussion

Overall, foreigners are a population that makes contact with the health care system primarily for physiological or accidental events. Their impact on the National Health System is limited (less than 3%), even less than their demographic share (about 5%). The observed variability among regions depends on the different levels of immigration.

The analysis of data from Lazio shows that immigrants utilise fewer health resources than the resident population, with a ratio 0.8 for acute care and 0.7 for day care. Critical areas have been identified in which the rate of hospitalisation is higher among immigrants than in the resident population. First, adult immigrants are more vulnerable to injuries, these being the main cause of hospitalisation among foreign males. Greater vulnerability to injuries might be related to poor living and working conditions. It is not possible to calculate the fraction of injuries that take place in the work environment. However, immigrants are often exposed to hazardous work, have insufficient training and high mobility, and experience the stress of adaptation to different work environments. Surveys of injuries at work conducted in Italy have suggested a higher risk for immigrants [21,22]; and studies conducted in various European countries have reported that migrant workers have higher rates of occupational accidents and consequent disability than native workers [23,24].

Second, age-specific rates show that immigrants are more frequently hospitalised for infectious diseases, particularly HIV and tuberculosis. These results might partly be related to the presence of the various specialised health institutes for infectious diseases in Lazio, which draw cases to the region. Nevertheless, analysis of more recent data in Lazio shows a decreasing trend in the numbers and percentages of discharges for infectious diseases among immigrants; and for AIDS, a national study shows that incidence of this disease among foreigners has been decreasing in recent years, which reflects the trend among Italians [25].

Third, alarming results emerge on reproductive health for women from LDCs. The incidence of induced abortions is very high among immigrant women, especially between the ages of 18 and 29 years. This result is in line with national data that show an age-standardised induced abortion rate three times that of residents [26]. Possible explanations are the inability or difficulty of immigrant women in controlling and planning their own reproductive life, and circumstances that may limit their opportunity to carry through a pregnancy, such as poor living conditions, social and work instability, and the lack of social support.

Finally, we observed higher rates for Symptoms, Signs and Ill-Defined Conditions, which may indicate cultural and linguistic difficulties in the clinician-patient relationship and may result in low quality assistance to immigrants. Alternatively, this group may suffer psychosomatic disturbances that are difficult to define.

The results for the population under 18 years old confirm that immigrants seem to be generally healthy, and admission for major causes, respiratory diseases and injuries and poisoning, are similar for foreigners and Italians, particularly when account is taken of the difference in the age structure of the populations. The only important differences are for infectious and parasitic diseases, with a higher proportion among immigrant youths. Immigrants also have a longer duration of stay in comparison to Italians (6.7 vs 4.6 days).

The general picture emerging from this analysis is similar to that of other Italian studies. One study conducted in Rome reports deliveries and injuries as the most frequent causes of hospitalisation [27]; another finding is a larger diffusion of tuberculosis, trauma, and pregnancy among immigrants in Turin [28]; other analyses report a lower percentage of hospitalisations for immigrants compared with Italians [15,16,29]. Some international studies also show a lower utilisation of hospital resources by immigrants compared with the native population [10], or a lower use of specialised health care [9], although comparison with other countries is difficult due to different histories of immigration, and to different health and social policies.

Our findings suggest the persistence of the so-called healthy migrants effect [30] – according to which the healthiest and youngest people choose to go abroad in search of better living conditions – as the majority of discharges are not due to imported diseases. However, this effect may slowly decline as a consequence of both forced migration and displacement, and family reunions, which contribute to the social stability of the foreign group, but at the same time may weaken the average health status of the migrants on arrival. The health condition of the migrant population on arrival may be subject to rapid deterioration due to lifestyle changes or prolonged exposure to risk factors, such as the difficulty of integrating with the social fabric of the host country, poverty and discrimination with regard to access to social and health services. The increasing number of discharges for chronic diseases observed in recent data, in particular for cardiovascular diseases and tumours, suggests that this deterioration may already be in progress.

The observed under-utilisation of hospital resources, in particular day care, could, at least in part, suggest administrative, linguistic, and cultural barriers to health care access. Such barriers have also been reported for migrants and ethnic minorities in different European countries [7]. Furthermore, racism and discrimination within the health services have been reported as an additional barrier [31], although not specifically in Italy. It should be noted that, since in Italy free hospitalisation is guaranteed to the entire population without distinction, legal and financial barriers should not be numbered among the main causes of reduced access to hospital care. The social frailty of migrant groups appears to be the likely trigger for some critical health conditions. In contrast to the healthy migrants effect, there is also evidence that risk factors expose migrant populations to a substantial burden of disability later in life, the so-called exhausted health effect [6].

Different considerations relate to the validity of the information on citizenship, the key variable used to identify immigrants. It was introduced into the regional archive in the year 2000 and its validity has not been demonstrated either by specific studies or by its use. This could imply misclassification and biased results. Immigrants are probably more likely to be misclassified as Italians than vice versa. In this case, the effect of misclassification would be to underestimate the number of immigrants receiving hospital treatment, with the risk of both biased rates and biased association measures. Other flaws in the study may be related both to discharge and population data: due to illegal immigration, we were not able to identify the illegal immigrant population (foreigners without a stay permit) among patients, and thus the immigrant population at risk was underestimated. However, when we recalculated age-and gender-standardised rates using an alternative method of standardisation and taking into account the estimate of irregular immigrants (see endnote 3), we did not observe important differences. In addition, the comparison group (i.e. the resident population) includes foreign residents. However, the comparison population can be considered very similar to the Italian population resident in Lazio, since in 2000 immigrants still represented a small part (4%) of the total. Despite this, the considerable differences in hospitalisation patterns observed between immigrants and the resident population, and the consistency of the results with those reported in other studies lend some support to our results.

A minor limitation may be that we did not measure the confounding effect of socio-economic level because of its probable low validity, in particular when used for immigrants, even though it is usually considered as a confounder in studies concerning such groups. However, evidence has been reported that both migration status and low social position are independent risk factors associated with poor health [32,33] or lower utilisation of specialised health services [34].

It is clear that an analysis of hospital discharge records cannot provide a comprehensive picture of the health needs and health care of the immigrant population. However, the results of such an analysis should be sufficient to identify the more important differences that exist between immigrants and native population. These results suggest that adequate strategies of health prevention and social promotion should be planned for a multi-ethnic population in Italy. Accessibility to health services for immigrants may need to be improved; their special health needs should be identified and acknowledged and steps should be taken by health authorities to ensure that the effects of administrative, cultural and linguistic barriers are minimised. Finally, as the immigrant population becomes a more substantial part of the whole Italian population, it will be necessary to improve the quality of the information collected, in order to identify more precisely the differences in health needs, access and health care, between the immigrants and the native Italians.

## Endnotes

### 1. Classification of foreign countries

In the year 2000, according to the Italian National Institute for Statistics, countries of the European Union (15 countries), as well as Andorra, Vatican City, Iceland, Liechtenstein, Malta, Monaco, Norway, San Marino and Switzerland in Europe; Canada and the United States in America; Oceania; and Japan and Israel in Asia were considered Developed Countries. All the other foreign countries were classified as Less Developed Countries [1].

### 2. Health care system in Italy

Italy's health system is a regionally-based national health service, which provides universal coverage free of charge for hospital care and with co-payments for ambulatory and pharmaceutical care [35]. Immigrants legally living in the country have the right to join the National Health Service. Free access to a package of essential health services (i.e. emergencies, maternal and child clinics, compulsory vaccinations, hospital and ambulatory care for conditions which could represent a severe long-term health risk if left untreated) is guaranteed by a national act also to irregular immigrants, without duty to notify to immigration authorities. Referral from a general practitioner is needed to access specialised and pharmaceutical care but not for urgent hospital care. Regional borders represent barriers for primary care but not hospital care.

### 3. Indirect-inverse standardisation (derived by Professor John Osborn)

The commonly used methods of standardisation, known as the "direct" and "indirect" methods are used to compare risks, for example mortality rates (or in this analysis, hospital discharge rates), when it is required to eliminate the effect of a confounding variable, for example age.

Let *N*_*i*_, *D*_*i *_and *Q*_*i *_represent the number exposed to risk, the number of deaths and the risk observed in the standard or referent population (Italians) in age group *i*. Similarly let *n*_*i*_, *d*_*i *_and *q*_*i *_refer to these values in the index population (immigrants). Let *N *= *∑N*_*i*_, *D *= *∑D*_*i*_, *n *= *∑n*_*i*_, *d *= *∑d*_*i*_, and *Q *= *D*/*N *and *q *= *d*/*n *be the crude rates in the standard and index population respectively.

In the direct method, a standard population is defined and the age-specific index rates are applied to the corresponding age-specific standard populations to determine the expected number of deaths. The ratio of the total number of expected deaths to the total of the standard population is the directly standardised rate. The ratio of the directly standardised rate to the crude rate is known as the comparative mortality index (CMF). The CMF can be shown to be the weighted average of the age-specific rate ratios using the number of deaths in the standard population, *D*_*i*_, as weights.



In the indirect method, standard age-specific rates are defined and these rates are applied to the corresponding age-specific index populations to obtain the expected deaths in the index population. The ratio of the observed total number of deaths in the index population to the sum of the expected number is known as the standardised mortality ratio (SMR). As before, this standardised ratio can be expressed as the weighted average of the age-specific rate ratios, but in this case the weights are the expected numbers of deaths, *Q*_*i*_*n*_*i*_.



Neither of these two methods can be applied in the situation in which the age-specific distribution of the index population is unknown, as is the case with the immigrants in the present analysis. However, if the total of the immigrant population, *n*, is known, standardisation is still possible using a method we have called indirect-inverse standardisation.

Taking the Italian population rates as the standard, the expected number of immigrants in age group *i*, can be calculated by dividing the observed number of discharges of immigrants *d*_*i*_, by the age-specific rates in the Italian population *Q*_*i*_. The ratio, *R*, of the total expected number of immigrants to the observed total is a weighted average of the age-specific rate ratios with weights equal to *n*_*i *_because:



The standardised rate can be estimated by *QR*.

The standard error of this standardised ratio can be derived assuming that *d*_*i *_is either a binomial variable (in the case that *q*_*i *_is a proportion, for example a prevalence) or a Poisson variable (in the case that *q*_*i *_is a rate, for example an incidence).

If *d*_*i *_is binomial, the estimated variance of the standardised ratio is:



and SE(R) is the square root of this quantity.

Clearly if, as in the immigrant example, *n*_*i *_and *q*_*i *_are unknown, *n*_*i *_can be estimated by *d*_*i*_/(*RQ*_*i*_) and *q*_*i *_can be estimated by *RQ*_*i*_.

If *d*_*i *_is Poisson, the estimated variance of the standardised ratio is:



and SE(R) is the square root of this quantity.

### 4. Comparison rates

The total regional hospitalisation rates relate to the resident population, which includes both Italian and foreign residents in Lazio, both in the numerator and denominator. This choice is because the available sources of resident population data, differently from the "stay permits" data, do not allow differentiation between Italians and foreign citizens by age. Therefore, it is not possible to calculate age-standardised and age-specific rates only for Italian residents. Thus, the two populations compared partially overlap and this could cause an underestimate of the differences between them.

### 5. Estimate of irregular immigration

The best existing estimates of irregular immigration are those produced by the Episcopal organisation Caritas, which publishes annually a dossier on immigration. According to Caritas[36], the people living illegally in the country could be about 20% of the total number of immigrants in Italy. Unfortunately, the age distribution of this proportion is unknown, therefore it cannot be used to calculate age-specific rates.
